# The Cumulative Effect of Multilevel Factors on Myopia Prevalence, Incidence, and Progression Among Children and Adolescents in China During the COVID-19 Pandemic

**DOI:** 10.1167/tvst.11.12.9

**Published:** 2022-12-14

**Authors:** Yanhui Dong, Catherine Jan, Li Chen, Tao Ma, Jieyu Liu, Yi Zhang, Qi Ma, Panliang Zhong, Yi Song, Jun Ma, George C. Patton, Susan M. Sawyer

**Affiliations:** 1 Institute of Child and Adolescent Health, School of Public Health, Peking University, Beijing, China; 2 Centre for Eye Research Australia, Department of Ophthalmology, Royal Victorian Eye and Ear Hospital, East Melbourne, Melbourne, Australia; 3 School of Medicine, Dentistry and Health Sciences, University of Melbourne, Parkville, Victoria, Australia; 4 Lost Child's Vision Project, Sydney, New South Wales, Australia; 5 Department of Paediatrics, Faculty of Medicine, Dentistry and Health Sciences, University of Melbourne, Parkville, Victoria, Australia; 6 Murdoch Children's Research Institute, Parkville, Victoria, Australia; 7 Centre for Adolescent Health, Royal Children's Hospital, Parkville, Victoria, Australia

**Keywords:** myopia, online learning, school, predictors, children and adolescents, prevention

## Abstract

**Purpose:**

To estimate the effects of school closures and associated lifestyle changes on myopia in Chinese children and adolescents during the coronavirus disease 2019 (COVID-19) pandemic.

**Methods:**

Two cross-sectional surveys recruited 14,296 Chinese students aged 7 to 18 years in November 2019 and June 2020 from which an open cohort study (nested queue design) was derived and used to assess myopia prevalence, incidence, and progression rates (defined as students with progression in myopia severity at the second survey wave among those with myopia at baseline). The severity of myopia was determined by measurements of visual acuity (<5.0) and noncycloplegic refraction (spherical equivalent <−0.50 diopters). Twenty-three myopia-influencing factors were divided into three categories: eye-use habits, lifestyle, and family and subjective factors. Responses to each of these 23 factors were labeled as either positive or negative options and then combined to generate a comprehensive score.

**Results:**

Boys and girls were equally represented (50%) and had the same average age (11.5 years) at each wave. The myopia prevalence increased from 48.2% to 60.0%, with 27.1% myopia incidence and 13.2% myopia progression rates for Chinese children and adolescents. Each of the 23 factors was associated with myopia prevalence but had no significant effect on myopia incidence or progression. However, these 23 factors had a cumulative effect on myopia risks; higher scores were associated with more positive factors and lower risk ratios of myopia and vice versa. Except for the progression rate, the myopia prevalence and incidence and risk ratios decreased with higher comprehensive scores.

**Conclusions:**

School closures during the COVID-19 pandemic increased the risk of myopia in Chinese children and adolescents due to the accumulation of poor eyesight habits, unhealthy lifestyles, and excessive screen time.

**Translational Relevance:**

Rather than focusing on single risk factors for myopia, future myopia prevention strategies should focus on integrating multiple comprehensive approaches across schools, families, and communities.

## Introduction

The world has been challenged by the novel coronavirus disease 2019 (COVID-19) pandemic that first appeared at the end of 2019.^1^ After its initial outbreak, countries and cities around the world took unprecedented steps to respond to this public health crisis, including introducing travel bans and stay-at-home orders, as well as promoting physical and social distancing. Consistent with these measures, many countries closed schools, and almost all school-aged children and adolescents shifted to home-based learning.^2^ In April 2020, at the peak time of school closures, at least 1.5 billion children and adolescents in over 200 countries were affected by these policies.^3^ From the end of February to early May 2020, almost all schools in China were closed. Similarly, in the United States, school closures began in mid-March 2020 and lasted until at least the end of the year.^4^ School closures have substantially disrupted the lives of children, adolescents, and their families, with far-reaching consequences on student learning, physical health, and emotional well-being.^5^^,^^6^

During the first half-year of 2020 in China, home-based online learning became the main mechanism for school-aged children and adolescents to engage with formal education. Beyond direct impacts on learning, school closures and the greater time spent by students online during the COVID-19 pandemic have also been widely anticipated to contribute to lifestyle changes such as increased sedentary time, insufficient time spent outdoors, decreased physical activities, less favorable diets, irregular sleep patterns, and high intensity of close work activities (e.g., reading, writing, and looking at the screens of electronic devices).^7^ Some of these behaviors could lead to or intensify myopia,^5^^,^^8^^,^^9^ with much discussion by Chinese experts who have labeled the set of health risks from school closures as “quarantine myopia.”^5^

In 2020, beyond China, widespread concerns have been raised about the impact of the COVID-19 pandemic on child and adolescent myopia. Some surveys reported change in myopia prevalence during the COVID-19 pandemic and the potential influence of behavioral factors.^7^^,^^10^^–^^16^ However, these studies were variably limited by being small in scale or cross-sectional in design, and the effects of various confounders could not be excluded such as individual lifestyle characteristics, intervals between school closures, or age effects. To our knowledge, there has been no prospective analysis using original data at a national level to investigate the current situation of myopia and its changes in children and adolescents during the COVID-19 pandemic, particularly within the context of school closures and highly intensive online learning.^5^^,^^17^^,^^18^ Furthermore, no study to our knowledge has explored the association of different patterns of behaviors during school closures with myopia in children and adolescents. Using nationally matched longitudinal data across the COVID-19 pandemic in China in November 2019 and June 2020, this study describes the prevalence, incidence, and progression rate of myopia and identifies factors influencing myopia occurrence and progression among children and adolescents during the COVID-19 school closures.

## Method

### Study Design and Participants

**Figure 1.**
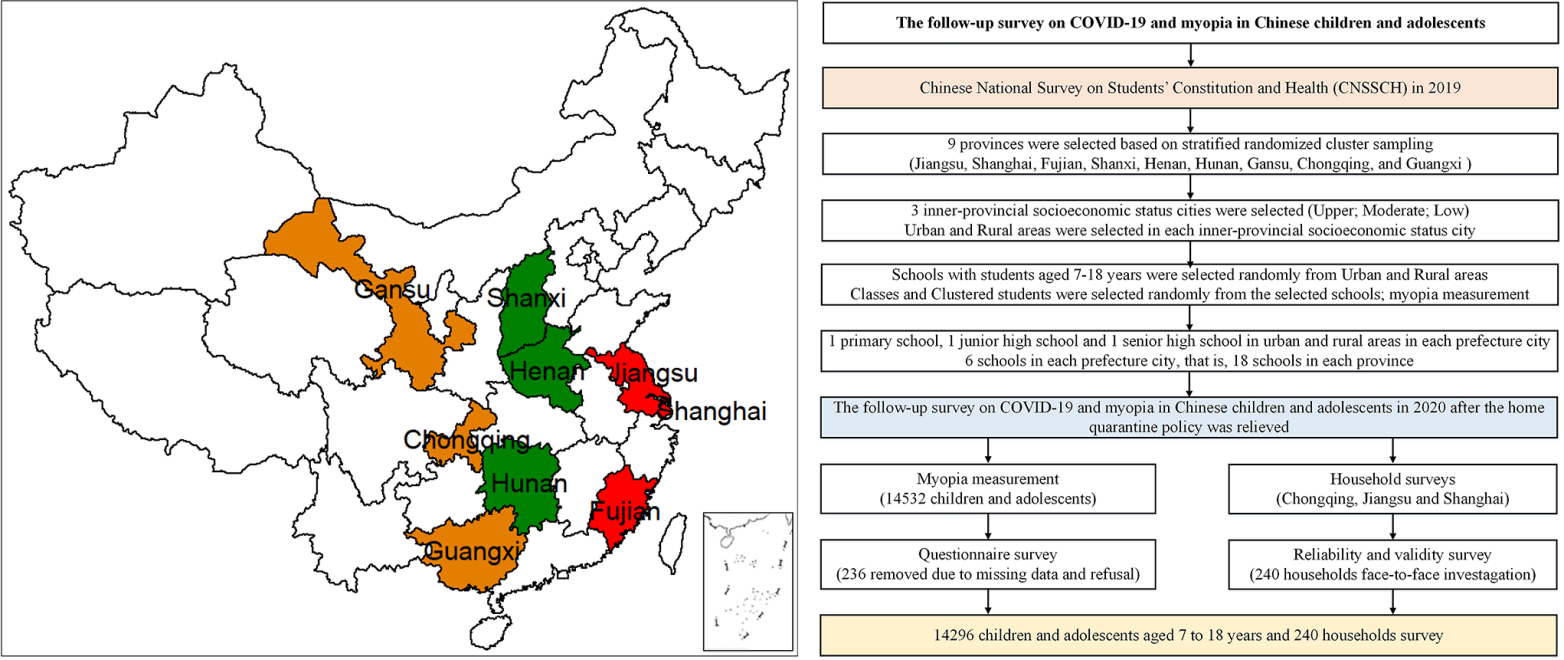
Geographical distribution of nine provinces of myopia investigation from 2019 to 2020 before and after COVID-19 pandemic (A) and the flow chart of data (B). Three regions of China were represented in A, such as East (*red*), Central (*green*), and West (*orange*).

An open cohort study (nested queue design) was achieved by extracting data from two cross-sectional surveys, the first in November 2019 and the second in June 2020, using a multistage stratified cluster sampling design. These two time points corresponded to the time before and after national school closures from the COVID-19 pandemic in China. The first survey, physically conducted in schools before the COVID-19 pandemic, built on the Chinese National Survey on Students’ Constitution and Health; its sampling and investigation procedures have been described previously.^19^^,^^20^ On the basis of the results from this survey, nine provinces were randomly selected by the level of myopia (high, medium, and low) to complete a follow-up survey in June 2020, soon after all of the survey schools had reopened. The sample distribution of eastern, central, and western regions was taken into account and included Jiangsu, Shanghai, Fujian, Shanxi, Henan, Hunan, Gansu, Chongqing, and Guangxi (Fig. 1). A total of 236 participants (1.6%) were excluded from analysis due to missing data or lack of consent for follow-up, which resulted in data from 14,296 (98.4%) students aged 7 to 18 years (50.02% boys vs. 49.98% girls) included in the final analysis of the two waves. Balanced samples of participants were collected from the nine provinces by region (eastern, central, western), which included urban and rural areas. The mean (SD) age was 11.5 (3.2) years in both boys and girls, and the proportions within the sample by age group were 62.5% for 7- to 12-year-olds (primary school), 20.2% for 13- to 15-year-olds (middle school), and 17.3% for 16- to 18-year-olds (high school). Before the survey, children and their parents were asked to provide signed, informed consent. The headteacher of each class explained the purpose of the study to parents who were provided with relevant information regarding the questionnaire. Consent forms were collected the next day, and only those students who agreed to participate in the study were asked to complete the questionnaire and physical examination. The study was approved by the Medical Research Ethics Committee of the Peking University Health Science Center (IRB00001052-21001).

### Measurement and Definition

The same vision measurements were conducted for both waves. For this study, we defined myopia using a combination of unaided distance visual acuity (VA) and noncycloplegic refraction (spherical equivalent <−0.50 diopters [D]), in accordance with the myopia definition for population surveys set by the National Health Commission of China and referred to by the International Council of Ophthalmology in cooperation with the World Health Organization and the International Agency for the Prevention of Blindness.^21^^,^^22^ Because of the large sample size, conducting cycloplegic refraction (the criterion that is standardly used to diagnose myopia) was not feasible. Unaided distance VA should be measured from a standard distance (5 m), using a standard chart of Illiterate E with a white background. The reduced VA was defined as the measurement value of less than 5.0 in either eye. Myopia was defined when participants had reduced VA and had a spherical equivalent (SE) less than −0.50 D using noncycloplegic and subjective refraction. Mild myopia was defined as the equivalent ball mirror (SE) less than −0.50 D but greater than or equal to −3.00 D, moderate myopia was defined as less than −3.00 D but greater than or equal to −6.00 D, and severe myopia was defined as less than −6.00 D. We defined myopia progression at the second wave of the study in 2020 if an individual student's VA and SE values had progressed to a more severe group among those with myopia at the 2019 baseline survey. The myopia progression rate was defined as the number of children who had myopia at the first wave whose myopia group had progressed between the first and the second waves divided by the number of those with myopia at baseline. Myopia incidence was defined as the number of children with newly detected myopia in the second wave of the survey divided by the number of those without myopia at baseline, as shown in Supplementary Figure S1.

### Questionnaire Survey

Before the survey, all survey supervisors attended a standardized training session. These supervisors attended each class with those students who agreed to participate in the survey when the survey was undertaken and were available to ensure that children and adolescents understood each question, had the opportunity to ask any questions, and provided guidance to students during the survey if needed. Parents were requested to assist younger children to complete the questionnaire at home and return the completed questionnaire the next day. All questions were checked for logic by the investigators.

In the survey, questions were asked that were based on the classification of the major factors affecting children's myopia in the COVID-19 pandemic by Navel and colleagues.^23^ Beyond general information, the questionnaire covered 23 myopia-influencing factors from three categories: eye-use habits, lifestyle factors, and family and subjective factors (Supplementary Table S1). During data analysis, we then classified each survey question response options into two categories: a positive factors group and a negative factors group, according to international or national standards and the distribution of question response options. For example, if a behavior is beneficial to eye health or known to prevent myopia or reduce myopia progression, we categorized it as “positive.” Some variables had internationally accepted cutoff values around children and adolescents^24^; we adopted the cutoff points of these continuous variables, including for dietary behavior, physical activity, sleep duration, drinking behavior, and sedentary behavior. Two factors did not have uniform cutoffs, and we classified them based on similar variables with available reference cutoffs or the 50th percentile of the sample, such as the amount of moderate-to-vigorous physical activity time (MVPA) (we used the reference cutoff of 1 hour/day for the amount of outdoor time) and sedentary time (50th percentile of the sample). The detailed information of verifying the reliability and validity of the questionnaire is detailed in Supplementary Table S2.

### Statistical Analysis

Categorical variables were compared between different groups using chi-square tests. We used the Cochran–Armitage trend test to calculate the *P*_trend_ values. The adjusted risk ratios (RRs) were calculated after adjusting for age, sex, area (urban/rural), province, and the clustering effect of schools to assess the separate effect of each factor on myopia prevalence, incidence, and progression rate. Based on the longitudinal design of our study, the adjusted RRs for myopia incidence and progression rate were calculated by the postestimation command, adjrr, that runs a logit model with a binary outcome.^25^ When the prevalence of events is high (>10%), using the odds ratio to describe the strength of the association would overestimate the association between each factor and childhood myopia.^26^ In cross-sectional studies, the log-binomial model more accurately assesses the strength of the association between the factors and outcome variables, expressed as an RR index. In our model, the province and region were adjusted in the analyses, but the cluster effects for school were added to the regression model. The three regions (East, Central, and West) were categorized according to the geographical standards from the Chinese National Bureau of Statistics.^27^

To evaluate the overall effect of all the factor scores on myopia, we scored the responses and assigned 1 point to each positive factor and –1 point to each negative factor. We then calculated the total score from the 23 different questions, which comprehensively spanned the three categories. We divided the comprehensive scores into eight groups according to the distribution of comprehensive scores to obtain a similar size in each group. We then calculated the adjusted myopia prevalence, incidence, and progression rates and their RRs in each comprehensive score group with the reference group of comprehensive scores as “0.” This approach was used to verify the association between the comprehensive score and myopia risk. As the relationship between comprehensive score and myopia (prevalence, incidence, and progression rates) has not been shown to be either linear or nonlinear, we took a conservative approach and used both linear and nonlinear models. To further determine the nonlinear associations between the total scores of comprehensive influencing factors and myopia risks, as well as to quantify differences in the associations between different subgroups, we employed generalized additive models to calculate the nonlinear fitting curve of the relative risk values of myopia prevalence, incidence, and progression rates with the total comprehensive scores (based on the median scores of “0”) calculated in boys and girls, for the three age groups, and the different regions. Because myopia/no myopia is binary, we used logit family to draw the associations. The confounding factors that we added to the models of generalized additive models, logit model, and log-binomial model included age, sex, region and province, and the cluster effect of schools.

To understand the contribution of each of the three categories of factors to the risk of myopia status, occurrence, progression, and population-attributable risks (PARs, percentage) with corresponding 95% confidence intervals (CIs) were estimated based on asymptotic approximations that derived PAR using the formula provided by Greenland and Drescher.^28^ Calculation of PAR implies a theoretical causal relationship. Models were conducted based on the logistic regression model using the aflogit module for Stata adjusting for age, sex, region, and province. The theoretical myopia prevalence, incidence, and progression rates were assessed using the actual rates multiplied by 1 – PAR% if the 23 related influencing factors were eliminated or controlled, which reflected the intervention effects on myopia if the three category factors or all 23 factors were theoretically averted in children and adolescents.^29^ All analyses were performed with Stata V.16 software (StataCorp, College Station, TX, USA).

## Results

### Myopia Changes During School Closures

**Figure 2.**
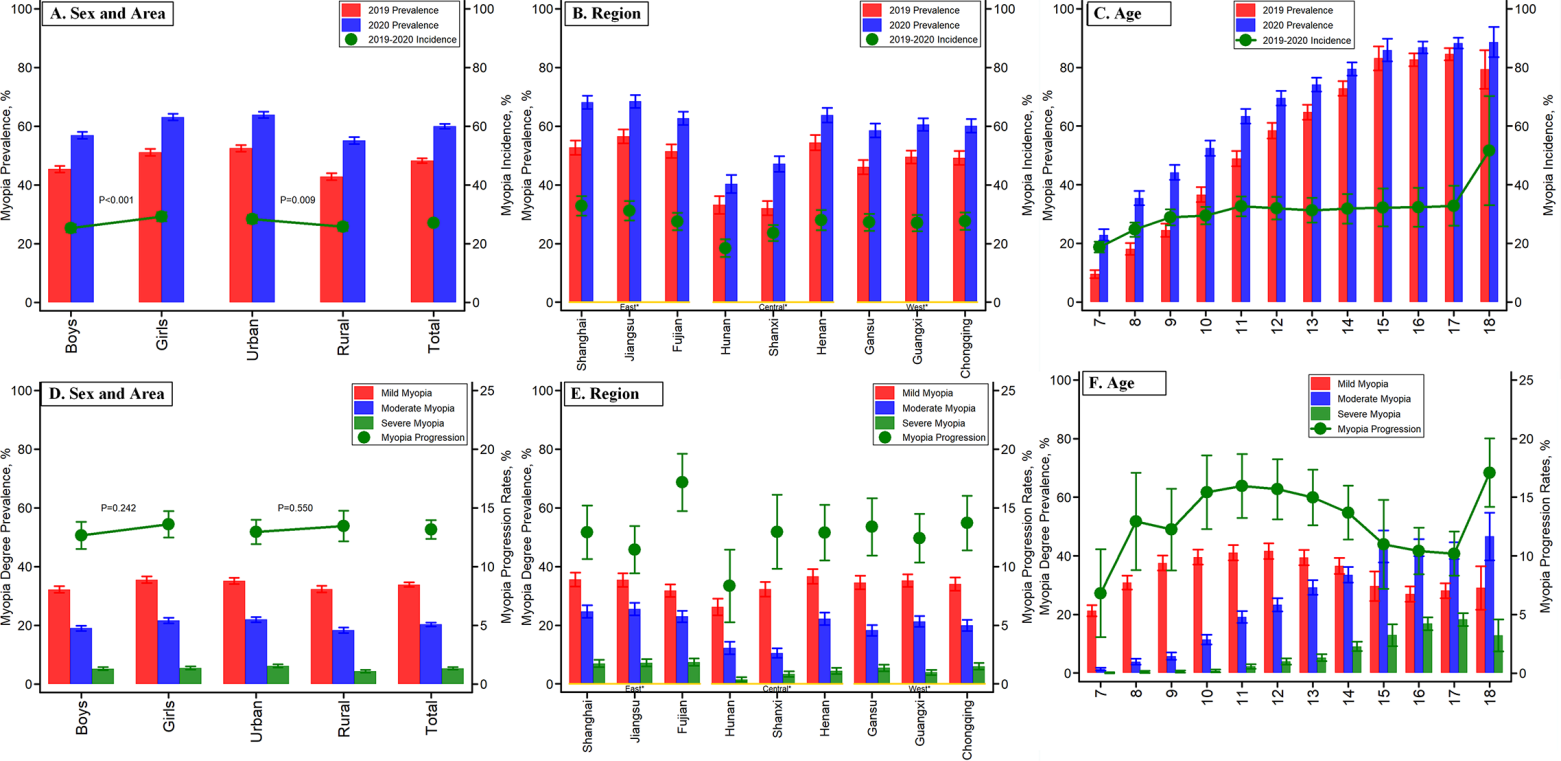
Myopia prevalence, incidence, severities of myopia prevalence, and progression rates among children and adolescents by sex, area, region, and age from 2019 to 2020 during the COVID-19 pandemic.

In 2019, the myopia prevalence of children and adolescents was 48.2%, which increased to 60.0% in 2020, an 11.8 percentage point increase after school closures. In both waves, the myopia prevalence in girls was higher than in boys (51.1% vs. 45.4% in 2019; 63.1% vs. 56.9% in 2020) with a similar increase in girls (12.0 percentage points) and boys (11.6 percentage points). The myopia incidence during school closures was higher in girls than in boys (29.19% vs. 25.3%), and the myopia progression rate was also higher in girls than in boys (13.6% vs. 12.7%) (Fig. 2, Supplementary Tables S3 and S4).

### Myopia-Related Factors and Their Effects on Myopia During School Closures

As shown in Table and Supplementary Table S1, eye-use habits during the lockdown differed from those during ordinary school life. Among the daily positive lifestyle factors, the proportion of participants spending ≥1 hour per day outdoors, MVPA, or walking reached 71.1%, 38.7%, and 30.7%, respectively. As for the family and subjective risk factors, about 30% of participants had a family history of myopia, and about 40% reported having experienced blurred vision or eyestrain during the lockdown.

**Table.** Demographic Characteristics, Myopia Status, and Myopia-Influencing Factors of Students Aged 7 to 18 Years From 2019 to 2020, Before and After the COVID-19 Pandemic

	Boys		Girls		Total	
Variable	*n*	%	*n*	%	*n*	%
Sample in 2019 and 2020	7151	50.0	7145	50.0	14,296	—
Average age, mean/SD, y	11.5	3.2	11.5	3.2	11.5	3.2
Urban	4013	56.1	3984	55.8	7997	55.9
Rural	3138	43.9	3161	44.2	6299	44.1
7–12 years	4467	62.5	4466	62.5	8933	62.5
13–15 years	1438	20.1	1448	20.3	2886	20.2
16–18 years	1246	17.4	1231	17.2	2477	17.3
Myopia prevalence						
2019	3245	45.4	3651	51.1	6896	48.2
2020	4071	56.9	4510	63.1	8581	60.0
2019–2020 difference	826	11.6	859	12.0	1685	11.8
2020 myopia incidence	987	25.3	1020	29.2	2007	27.1
2019 myopia prevalence (proportion)						
Normal	3945	55.2	3544	49.6	7489	52.4
Mild	1762	24.6	1974	27.6	3736	26.1
Moderate	1144	16.0	1327	18.6	2471	17.3
Severe	300	4.2	300	4.2	600	4.2
2020 myopia prevalence (proportion)						
Normal	3156	44.1	2722	38.1	5878	41.1
Mild	2280	31.9	2508	35.1	4788	33.5
Moderate	1344	18.8	1529	21.4	2873	20.1
Severe	371	5.2	386	5.4	757	5.3
Myopia progression rate	406	12.7	490	13.6	896	13.2
Eye-use habit factors						
Online courses (yes)	5691	79.9	5695	79.8	11,386	79.9
Online courses duration (≥5 h/d)	820	13.5	814	13.3	1634	13.4
Online courses break (yes)	6022	90.3	6136	91.4	12,158	90.8
Overlooking habit (yes)	4484	67.4	4502	67.3	8986	67.4
Screen time (≥2.5 h/d)	2737	39.9	2751	40.4	5488	40.1
Video games time (≥4 h/d)	538	8.0	272	4.1	810	6.0
Lying down watching (yes)	5104	71.7	5249	73.7	10,353	72.7
Dark watching (yes)	2802	39.3	3038	42.7	5840	41.0
Lifestyle factors						
MVPA time (≥1 h/d)	1910	42.5	1482	34.8	3392	38.7
Walking time (≥1 h/d)	1269	31.6	1196	29.8	2465	30.7
Sedentary time (≥5 h/d)	2062	50.1	2063	51.1	4125	50.6
Outdoor time (≥1 h/d)	4889	72.8	4611	69.3	9500	71.1
Eats sweets (often)	5658	79.5	6059	85.3	11,717	824
Drinks SSB (often)	5081	71.4	4907	69.0	9988	70.2
Eats fried food (often)	4775	67.1	4912	69.1	9687	68.1
Sleep duration (low)	2183	41.3	2141	40.7	4324	41.0
Family and subjective factors						
Father myopia (yes)	1929	27.1	2059	28.9	3988	28.0
Mother myopia (yes)	2149	30.2	2234	31.3	4383	30.7
Self-perceived visual loss (yes)	2337	40.1	2778	47.0	5115	43.6
Self-perceived eyestrain (yes)	2536	39.3	2963	45.5	5499	42.4
Sitting posture reminders (yes)	5843	82.1	5958	83.7	11,801	82.9
Desk brightness (no)	1953	27.8	2072	29.3	4025	28.5
Seat/height fit level (no)	606	8.9	615	9.0	1221	9.0

SSB, sugar sweetened beverages.

**Figure 3.**
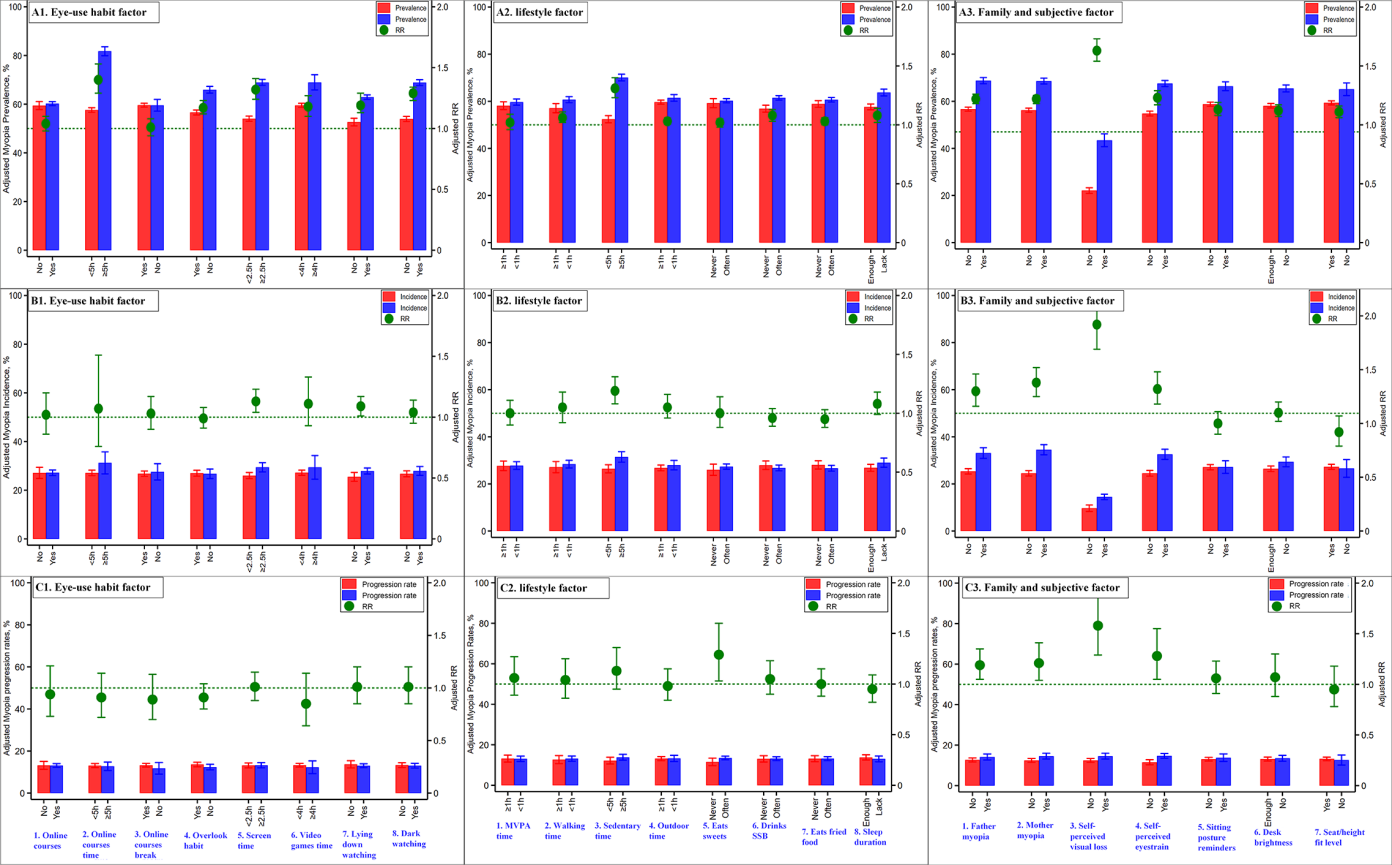
The comparison and risk ratios of adjusted myopia prevalence, incidence, severities of myopia prevalence, and progression rates in 23 different eye-use habits, lifestyle, and family and subjective factors among children and adolescents in school closures from 2019 to 2020 during the COVID-19 pandemic.

Children and adolescents with each positive factor within the three categories of eye-use habits, lifestyle factors, and family and subjective factors had a lower prevalence of myopia compared to those with negative factors from the same category. For example, the prevalence of myopia in children and adolescents who spent more than 5 hours daily on school online courses was 80.4%, which was higher than those who spent less than 5 hours (57.3%), a statistically significant RR of 1.40 (95% CI, 1.29–1.53). Similarly, almost all the negative factors within each of the 23 factors across the three categories were associated with a higher incidence of myopia in children and adolescents. With the exception of myopia family history and subjective blurred vision and eyestrain, none of the other 20 eye-use habit factors, whether negative or positive, had any significant effect on the progression of myopia during the school closures once myopia was present at the time of the baseline survey (Fig. 3 and Supplementary Table S5).

### Cumulative Effect of Influencing Factors on Myopia

**Figure 4.**
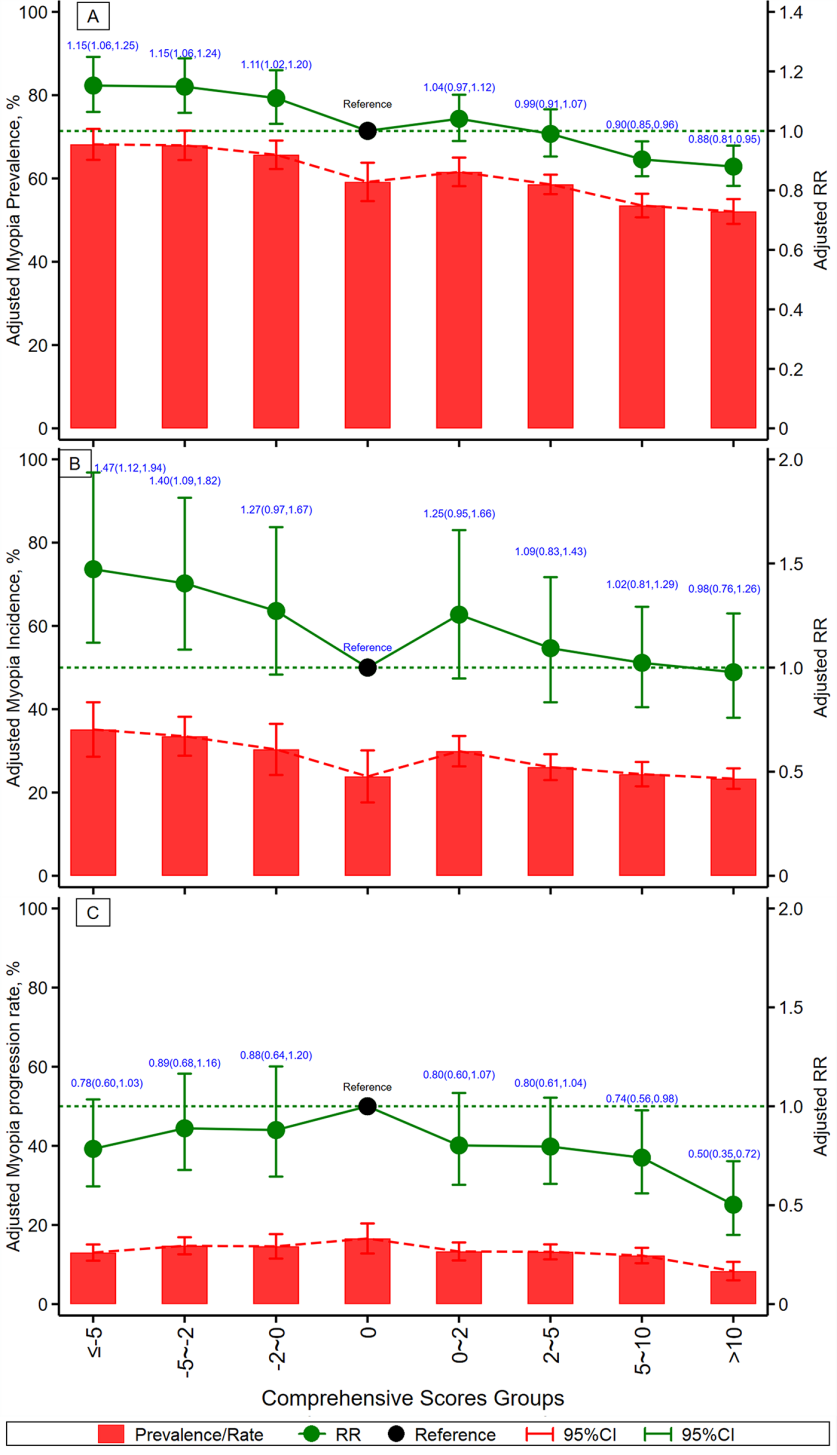
The change of adjusted myopia prevalence, incidence, and progression rates and its risks ratios by 8 comprehensive scores groups, based on 23 different eye-use habits lifestyle, family, and subjective factors. *Black dots* represent the reference group with comprehensive scores “0.”

**Figure 5.**
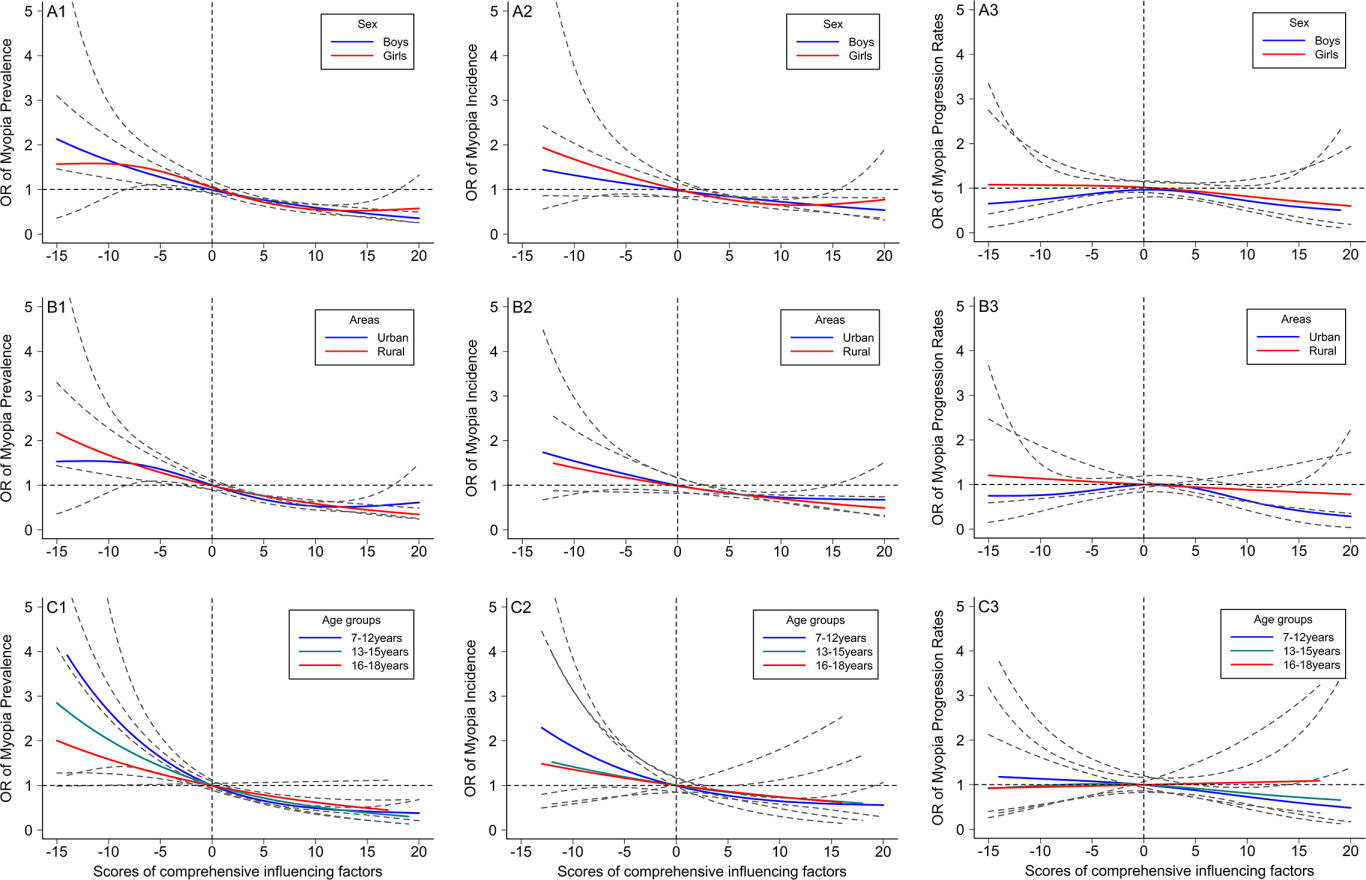
Comparison analysis between different subgroups for association between scores of comprehensive influencing factors and myopia prevalence, incidence, and progression rates. *Dotted gray lines* indicate 95% CIs. Generalized additive models were used to calculate the nonlinear fitting curve of the OR values of myopia prevalence, incidence, and progression rates with the scores of comprehensive influencing factors (based on the median scores of “0”). OR, odds ratio.

After integrating the positive and negative scores for each of the 23 factors, a comprehensive score of influencing factors was obtained for each child. The higher the score, the healthier the myopia-related factors for each child. Myopia prevalence and incidence and their RRs decreased with higher comprehensive scores. As shown in Figure 4, for example, the myopia prevalence steadily decreased from 68.2% in those with low scores (≤−5) to 52.1% in the group with high scores (>10), with significant RRs from 1.15 (1.06–1.25) to 0.88 (0.81–0.95). The myopia incidence similarly reduced from 35.1% to 23.3% with increasing scores, with significant RRs from 1.47 (1.12–1.94) to 0.98 (0.76–1.26; *P*_trend_ < 0.05; Figure 4 and Supplementary Table S6). As shown in Figure 5, a further nonlinear generalized additive model verified the same results whereby high comprehensive scores had a significant negative association with myopia prevalence and incidence, while low comprehensive scores had a significant positive association with myopia prevalence and incidence.

### Attribution of Cumulative Influences and the Theoretical Effect of Their Elimination

**Figure 6.**
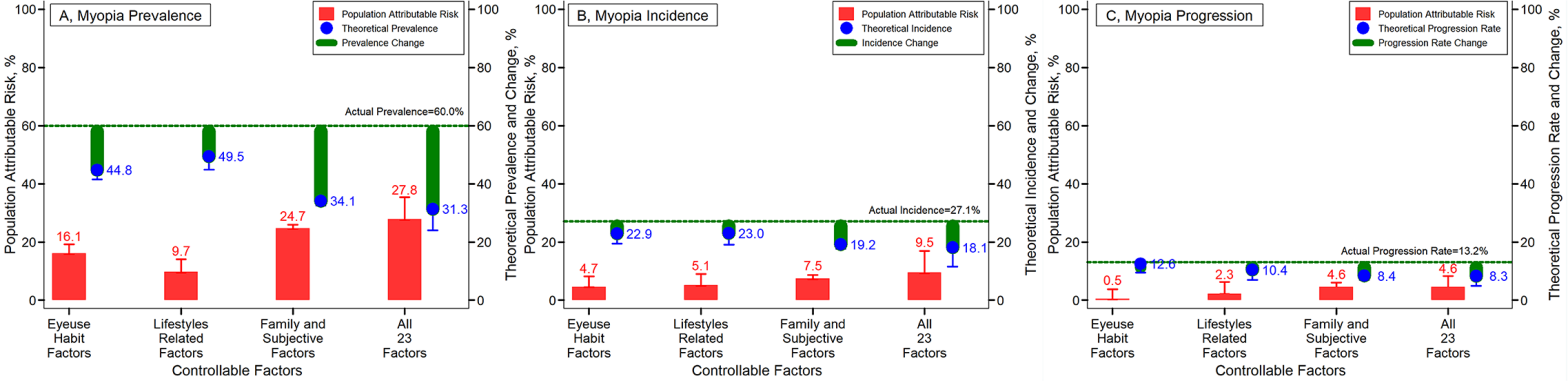
The population attributable risk and theoretical prevalence, incidence, and progression rates of myopia and its theoretical changes to actual levels of 23 potential influencing factors.

Figure 6 presents the PAR% for myopia from multiple-level influencing factors. It also shows the theoretical myopia prevalence, incidence, and progression rates if multiple-level influencing factors were eliminated by estimating PAR%. The PAR% for myopia prevalence for eye-use habit factors, lifestyle-related factors, and family and subjective factors were 16.1% (12.9%–19.3%), 9.7% (5.3%–14.1%), and 24.7% (23.4%–26.0%), respectively. Based on the actual myopia of 60.0%, the theoretical myopia prevalence reached up to 44.8% (41.5%–48.1%), 49.5% (44.9%–54.1%), and 34.1% (32.5%–35.8%) if these factors were separately eliminated from the three categories. Concurrent elimination of these factors within the three categories could theoretically reduce the myopia prevalence from 60.0% to 31.3% (24.0%–39.7%) due to the overall PAR% of 27.8% (19.9%–35.4%). Similarly, the myopia incidence and progression rates could theoretically be reduced to 18.1% (11.6%–27.1%) and 8.3% (5.0%–13.7%) from 27.1% and 13.2%, respectively, if all 23 factors were eliminated simultaneously (Supplementary Table S7).

## Discussion

This study used a nationally matched open cohort design to study the incidence of myopia and the factors influencing its incidence and progression in Chinese children and adolescents during school closures from the COVID-19 pandemic in 2019 and 2020. Our findings provide significant new evidence and update insights for myopia prevention in the context of the dramatic global shift to online learning for children and adolescents that occurred during this period. We found striking changes in the lifestyles of Chinese children and adolescents brought about by the COVID-19 pandemic, which have indeed had a significant impact on children's myopia development.

To contain the spread of the COVID-19 virus, consistent with World Health Organization intentions, students across the world overwhelmingly stayed at home, did not attend school physically, and limited many outdoor activities.^30^ Instead, many children and adolescents switched to online classes and remained largely indoors reading books, watching television, playing video games, or using computers, tablets, and smartphones to access learning, recreation, and play, as well as engage socially with peers.^17^ In our study, 80% of children and adolescents had continuous online classes during the early period of the pandemic, 50% reported spending more than 5 hours in sedentary activities each day, and 13% spent more than 5 hours daily on continuous online classes. Even in a casual home environment, 73% of children and adolescents reported watching electronic screens lying down, and 41% reported watching electronic screens in the dark. These behaviors far exceed the frequency of various unhealthy behaviors previously reported in the literature.^31^^,^^32^

Previous studies have reported that overuse of electronic devices and increased screen time will dramatically overstimulate accommodative effort induced by close working distances, and they represent a greater risk of myopia for children and adolescents.^33^ Interestingly, the COVID-19 pandemic prompted more than 38.7% of Chinese children and adolescents to engage in 60 minutes daily of MVPA, higher than previously reported in 2017 (34.1%).^34^ However, beyond that, the COVID-19 pandemic also appears to have greatly increased children's screen time. In comparison to a previous Chinese study from 2017, which found that 34.6% of 7- to 19-year-olds reported more than 2 hours/day of screen time,^34^ only 3 years later and in the context of the pandemic, we found that 40.1% of children and adolescents viewed screens more than 2.5 hours per day. By inducing an environment of intensive online learning, accompanied by high levels of recreational screen time, school lockdown policies in China during the COVID-19 pandemic provide strong evidence about the factors responsible for the rapid development of myopia.

Multiple individual factors have previously been shown to be associated with myopia in children such as intensive education, prolonged near work, and limited time outdoors.^35^^,^^36^ Yet consistent with prior research,^37^ we found that individual factors, such as eyesight habit factors, lifestyle-related factors, and family and subjective factors, had little significance on the incidence and progression of children's myopia during this period of school closures. One cohort study with 4 years of follow-up from Shanghai, China, found that only parental myopia, but not near work time, near work diopter time, outdoor activity time, or attending tutoring classes, was associated with myopia incidence or its progression in children.^37^ To our knowledge, we are the first to demonstrate the existence of the cumulative effect of multiple factors on myopia incidence and progression in children and adolescents. This is even more remarkable due to the relatively short period of follow-up, which encapsulated 9 months of school closures. We have shown that healthy lifestyle behaviors and eyesight habits, such as adequate sleep, healthy diet, regular physical activity, less screen and sedentary time, and less online study, demonstrate a cumulative benefit that results in lowering the myopia risk for school-aged children and adolescents. In the context of a 24-hour day,^38^ we note it is difficult for children and adolescents to concurrently achieve all 23 positive eyesight items. Yet it was common during school closures for students to reach 23 negative items. This information is important for considering approaches to prevention and control of myopia in children and adolescents. We have also shown that the theoretical control of the cumulative effect of multiple factors due to the COVID-19 pandemic could potentially have reduced myopia prevalence by 27.8%, myopia incidence by 9.5%, and myopia progression by 4.6% in Chinese children and adolescents. Notwithstanding genetic susceptibility, the present study also confirmed that these individually controllable behavioral factors are able to reduce myopia occurrence and its progression.

Our study has implications for policy making around myopia prevention and control. First, this study helps understand the effect of school closures and the shift to online learning and recreational screen time on myopia during the COVID-19 pandemic. The extreme example provided by the impact of the pandemic on student lifestyles may also have reflected individuals’ past lifestyles, with trends toward reduced time spent physically active outdoors being a feature of Chinese children and adolescents even before the COVID-19 pandemic.^39^^,^^40^ Given expectations that these sedentary lifestyles will become increasingly normative, affecting large proportions of children and adolescents, this study helps guide myopia prevention strategies in China as well as other countries that are increasingly burdened by a high prevalence of myopia. These strategies include policies that support comprehensive interventions within schools, families, and the community to lessen time spent learning online, decrease the burden of homework after school, reduce use of electronic devices (e.g., videogames), increase time outdoors through regular physical activity at school and in the community, develop eye health education (e.g., evidence-based eye use), and promote healthy eating and sleeping habits.^41^

Second, in the past, it has been difficult to assess the effectiveness of nationwide comprehensive preventive and control policies on children's myopia. In general, the effectiveness of a single intervention could only be assessed in a focused study that trialed outdoor physical activities or outdoor light intensity.^35^^,^^42^ Previous studies found that more time outdoors was associated with slower axial elongation in children who were not myopic but not in those with existing myopia. Similarly, some unhealthy habits, such as continuous and close distance reading, watching TV, and head tilting when writing, were associated with myopia in Chinese children.^43^ Our critical finding about the cumulative effect of individual factors suggests that prevention policies need to promote as many healthy behaviors as possible, even in the absence of individual factors having a significant impact on myopia over a short period. In China, a comprehensive national children's myopia management plan has been coordinated by eight central government bodies under the leadership of the State Council since 2018, which has focused on government policies, school-based prevention, and screening and treatment of myopia in both the health sector and the community.^44^^,^^45^ Our study reinforces the importance of such comprehensive interventions.

Third, regardless of the timing of school openings across the pandemic, online learning is likely to be an increasing feature of contemporary education and not just for tertiary students, as it had been prior to the pandemic in many parts of the world.^46^ A challenge for those responsible for educational and health policy is to ensure that health equity is not undermined due to the effect of family socioeconomic status, educational facilities, and health care quality on children's ability to access ideal environments for eye health that include bright rooms, comfortable desks and chairs, fun school playgrounds, high-quality face-to-face teaching (so that children and adolescents do not have to rely on online approaches), fast and reliable Internet connection, and supportive family environment. As a *Lancet* editorial noted, distance learning might be productive for some older children and adolescents, but certainly not all, and the digital divide created by inequities in access to technology and the Internet has deepened.^47^ This presents a set of new challenges around reducing health—and learning—inequities, with eye health reflecting this.

Our study has several strengths derived from its large nationally representative sample size, repeat measures within an open cohort, vision measurement that integrated various factors, and assessment of comprehensive myopia risks in children and adolescents. Three myopia indicators (prevalence, incidence, and progression rate) were used to evaluate the impact of the COVID-19 school closure period on myopia from an open cohort derived from careful comparison of year levels from two cross-sectional studies within the same regions and schools, an approach that helps to eliminate fixed effects from natural growth of children on myopia.

Our study also has several limitations. First, myopia was evaluated by combining the retroilluminated logMAR chart and noncycloplegic autorefraction followed by subjective refraction determining the refractive error, as recommended by the National Health Commission of China. Obtaining refractive error in the absence of cycloplegia may overestimate myopic power in children and adolescents, especially in children younger than 12 years.^48^^,^^49^ In addition, pseudomyopia is more prevalent in younger, more hyperopic children and might not be an independent risk factor for myopic progression even in cycloplegic settings.^50^ As the refractive error examination for cycloplegia is not suitable for large population studies such as ours, we suggest that these findings are consistent with a positive screen for myopia, rather than diagnosis. Previous studies have revealed that the sensitivity and specificity of our diagnostic methods in large population surveys reached 92.4% and 89.5%, and the area under the receiver operating characteristic curve combining the retroilluminated logMAR chart and noncycloplegic autorefraction reached 0.91.^51^ Thus, although this method is not adequate for diagnosis of myopia in a clinical setting, we consider it is an acceptable compromise for large population surveys. Considering global differences in background rates of physical activity and screen time prior to the pandemic; policy implementation among countries in relation to school closures, online learning, and physical distancing; and genetic risks, care should be taken in generalizing these findings outside the Chinese population, which has one of the highest rates of myopia in the world.^52^ Notwithstanding the hope that school closures from the COVID-19 pandemic might now be at an end, as the American Academy of Pediatrics pointed out in its guideline about school reopening in the United States in May 2020, communities should prepare for the possibility of repeated closures.^53^ Regardless, this study provides an important policy basis for myopia control within the current pandemic in China and beyond. Further limitations relate to the timing of the baseline survey (November 2019) and follow-up survey (June 2020), which were undertaken to coincide with school lockdowns in China. The different times of year may be a confounding factor, such as the timing of the exam season, with its expectations of more time spent studying. Recall bias cannot be excluded due to the nature of this questionnaire-based study. Finally, due to the large number of factors involved, there were some that did not have internationally referenced cutoffs for classification, many of which did not take into account the relative influence on myopia, which could be an important limitation.

In conclusion, around 8 months of school closures from November 2019 to June 2020 was associated with escalation of myopia among Chinese children and adolescents, whether measured by myopia prevalence, incidence, or progression rate, when compared to earlier assessment. A total of 23 factors, grouped into three categories of eyesight habit factors, lifestyle-related factors, and family and subjective factors, were associated with myopia prevalence, but individually few of these 23 factors had any significant impact on the incidence of myopia during the COVID-19 pandemic. In contrast, we found a cumulative effect of these factors on myopia in children and adolescents. Our findings provide additional evidence to support implementation of comprehensive intervention policies to prevent and control myopia among Chinese children and adolescents, which may be applicable to countries with similar myopia burdens.

## Supplementary Material

Supplement 1
