# Supplementary material for: The Cumulative Effect of Multilevel Factors on Myopia Prevalence, Incidence, and Progression Among Children and Adolescents in China During the COVID-19 Pandemic
Source: Transl Vis Sci Technol. 2022 Dec 14;11(12):9. doi: 10.1167/tvst.11.12.9 (PMC9756574; doi:10.1167/tvst.11.12.9)
Supplement: Supplement 1 [file tvst-11-12-9_s001.docx]

| **Table S1.** The details of questionnaires of 23 myopia influencing factors, samples, and adjusted myopia prevalence, incidence and progression rates, as well as their risk ratios in two question option (Positive v.s. Negative factor) during the COVID-19 epidemic | | | | | | | | | | |
| --- | --- | --- | --- | --- | --- | --- | --- | --- | --- | --- |
| **Questions** | **Options** | **Sample** | **Myopia Prevalence** | |  | **Myopia Incidence** | |  | **Myopia progression rates** | |
|  |  |  | **Prevalence** | **RR** |  | **Incidence** | **RR** |  | **Rate** | **RR** |
| **Eyeuse habits factors** | | | | | | | | | | |
| **Q1: (Online courses) Do you need to take online classes (computer, tablet or mobile phone, etc) every day when classes were not resumed during the COVID-19 epidemic?** | | | | | | | | | | |
| Positive: | No | 2874 | 59.4(57.8,61.1) | Ref. |  | 27.1(24.8,29.4) | Ref. |  | 13.2(11.3,15.1) | Ref. |
| Negative: | Yes | 11386 | 60.2(59.4,61.0) | 1.01(0.98,1.05) |  | 27.2(26.0,28.3) | 1.00(0.91,1.10) |  | 13.1(12.2,14.0) | 0.99(0.85,1.17) |
| **Q2: (Online courses time) The total amount of time you spent online during the COVID-19 epidemic without resuming classes? (Calculating according to the curriculum)** | | | | | | | | | | |
| Positive: | <5h | 10580 | 57.6(56.7,58.5) | Ref. |  | 27.1(26.0,28.3) | Ref. |  | 13.1(12.1,14.1) | Ref. |
| Negative: | ≥5h | 1634 | 81.8(79.9,83.6) | 1.42(1.38,1.46) |  | 31.2(26.7,35.7) | 1.15(0.99,1.34) |  | 12.8(10.8,14.8) | 0.97(0.82,1.16) |
| **Q3: (Online courses break) If classes are not resumed during the COVID-19 epidemic, will there be a break during each online class?** | | | | | | | | | | |
| Positive: | Yes | 12,158 | 59.6(58.8,60.4) | Ref. |  | 26.8(25.7,27.9) | Ref. |  | 13.3(12.4,14.1) | Ref. |
| Negative: | No | 1,227 | 59.5(57.1,62.0) | 1.00(0.9577,1.04) |  | 27.5(24.2,30.9) | 1.03(0.90,1.17) |  | 11.8(9.0,14.6) | 0.89(0.70,1.13) |
| **Q4: (Overlooking habit) Do you have the habit of looking far away during the online courses breaks** | | | | | | | | | | |
| Positive: | Yes | 8,986 | 56.6(55.6,57.6) | Ref. |  | 27.0(25.8,28.2) | Ref. |  | 13.6(12.6,14.7) | Ref. |
| Negative: | No | 4,356 | 65.9(64.5,67.3) | 1.16(1.13,1.20) |  | 26.7(24.8,28.7) | 0.99(0.91,1.08) |  | 12.4(11.1,13.7) | 0.91(0.80,1.04) |
| **Q5: (Screen time) How much time have you spent watching TV, computer, mobile phone and tablet (including surfing the Internet, watching movies, surfing the Web and playing games) every day during the COVID-19 epidemic?** | | | | | | | | | | |
| Positive: | <2.5h | 8,196 | 54.0(53.0,55.1) | Ref. |  | 26.0(24.8,27.2) | Ref. |  | 13.2(12.0,14.3) | Ref. |
| Negative: | ≥2.5h | 5,488 | 68.9(67.7,70.2) | 1.28(1.24,1.31) |  | 29.4(27.5,31.3) | 1.13(1.0,1.23) |  | 13.3(12.0,14.5) | 1.01(0.88,1.15) |
| **Q6: (Video games time) How much time per day have you spent playing video games during the COVID-19 epidemic?** | | | | | | | | | | |
| Positive: | <4h | 12,609 | 59.5(58.7,60.4) | Ref. |  | 27.2(26.1,28.3) | Ref. |  | 13.3(12.4,14.1) | Ref. |
| Negative: | ≥4h | 810 | 69.0(65.8,72.1) | 1.16(1.11,1.22) |  | 29.4(24.5,34.2) | 1.08(0.91,1.28) |  | 12.3(9.3,15.3) | 0.93(0.7176,,1.20) |
| **Q7: (Lying down watching) Have you been reading a book or watching a video (tablet or phone) while lying down during the COVID-19 epidemic?** | | | | | | | | | | |
| Positive: | No | 3,886 | 52.6(51.1,54.2) | Ref. |  | 25.5(23.7,27.3) | Ref. |  | 13.7(12.0,15.4) | Ref. |
| Negative: | Yes | 10,353 | 62.9(62.0,63.8) | 1.19(1.16,1.24) |  | 27.9(26.6,29.1) | 1.09(1.01,1.19) |  | 13.0(12.1,13.9) | 0.95(0.82,1.10) |
| **Q8: (Dark watching) Have you been often looking at your phone or tablet after turning off the lights during the COVID-19 epidemic?** | | | | | | | | | | |
| Positive: | No | 8,404 | 53.9(52.9,55.0) | Ref. |  | 26.7(25.5,28.0) | Ref. |  | 13.3(12.2,14.5) | Ref. |
| Negative: | Yes | 5,840 | 68.9(67.7,70.0) | 1.28(1.24,1.31) |  | 27.9(26.1,29.7) | 1.04(0.96,1.13) |  | 13.0(11.9,14.2) | 0.98(0.87,1.11) |
| **Lifestyles factors** | | | | | | | | | | |
| **Q1: (MVPA time) When classes did not resume during the COVID-19 epidemic, how much time did you spend doing moderate to high intensity physical exercise per day per week (7 days)? (Converting according to standard questionnaire)** | | | | | | | | | | |
| Positive: | ≥1h | 3,392 | 58.1(56.5,59.8) | Ref. |  | 27.7(25.7,29.7) | Ref. |  | 13.2(11.5,14.9) | Ref. |
| Negative: | <1h | 5,371 | 59.6(58.3,61.0) | 1.03(0.99,1.06) |  | 27.8(26.2,29.4) | 1.00(0.91,1.10) |  | 13.1(11.8,14.4) | 0.99(0.84,1.17) |
| **Q2: (Walking time) What is the average amount of walking time (and at least 10 minutes at a time) per day per week (7 days) during the COVID-19 epidemic when classes are not resumed?** | | | | | | | | | | |
| Positive: | ≥1h | 2,465 | 57.1(55.1,59.0) | Ref. |  | 27.1(24.7,29.5) | Ref. |  | 12.7(10.7,14.7) | Ref. |
| Negative: | <1h | 5,561 | 60.7(59.4,62.0) | 1.06(1.02,1.11) |  | 28.4(26.8,30.0) | 1.05(0.94,1.16) |  | 13.2(11.9,14.5) | 1.04(0.86,1.25) |
| **Q3: (Sedentary time) During the COVID-19 epidemic when classes are not resumed, how much time per day did you spend on sedentary time?** | | | | | | | | | | |
| Positive: | <5h | 4,029 | 52.4(50.9,53.9) | Ref. |  | 26.4(24.7,28.1) | Ref. |  | 12.2(10.5,13.9) | Ref. |
| Negative: | ≥5h | 4,125 | 70.1(68.7,71.5) | 1.34(1.29,1.39) |  | 31.5(29.2,33.7) | 1.19(1.08,1.31) |  | 13.8(12.4,15.3) | 1.13(0.95,1.36) |
| **Q4:(Outdoor time) What was your daily exposure to sunlight during the epidemic before classes resumed? (Time spent in direct sunlight)** | | | | | | | | | | |
| Positive: | ≥1h | 9,500 | 59.6(58.8,60.5) | Ref. |  | 26.8(25.6,28.0) | Ref. |  | 13.2(12.2,14.2) | Ref. |
| Negative: | <1h | 3,863 | 61.4(60.0,62.8) | 1.03(1.00,1.06) |  | 28.0(26.0,30.0) | 1.04(0.96,1.14) |  | 13.3(11.7,14.8) | 1.01(0.88,1.16) |
| **Q5:(Sweets eating) How many times a week did you eat sweets (including candies, cakes, chocolates, sweet soups, etc.) before classes resumed during the COVID-19 epidemic?** | | | | | | | | | | |
| Positive: | Never | 2,507 | 59.2(57.3,61.1) | Ref. |  | 26.0(23.6,28.4) | Ref. |  | 11.6(9.7,13.4) | Ref. |
| Negative: | Often | 11,717 | 60.2(59.3,61.1) | 1.02(0.98,1.05) |  | 27.4(26.2,28.5) | 1.05(0.95,1.16) |  | 13.5(12.6,14.4) | 1.17(0.98,1.39) |
| **Q6:(SSBs drinking) How many times a week did you drink sugar-sweetened beverages (including Cola, iced tea, nutrition express, etc.) before classes resumed during the COVID-19 epidemic?** | | | | | | | | | | |
| Positive: | Never | 4,242 | 56.9(55.4,58.3) | Ref. |  | 28.0(26.1,29.8) | Ref. |  | 13.0(11.5,14.6) | Ref. |
| Negative: | Often | 9,988 | 61.4(60.5,62.4) | 1.08(1.05,1.11) |  | 26.8(25.6,28.0) | 0.96(0.89,1.04) |  | 13.2(12.2,14.1) | 1.01(0.88,1.16) |
| **Q7:(Fried food eating) How many times a week did you eat fried food (such as deep-fried dough sticks, oil cakes, French fries, Fried chicken wings, etc.) before classes resumed during the COVID-19 epidemic?** | | | | | | | | | | |
| Positive: | Never | 4,546 | 58.8(57.4,60.3) | Ref. |  | 28.1(26.3,29.9) | Ref. |  | 13.1(11.7,14.6) | Ref. |
| Negative: | Often | 9,687 | 60.7(59.7,61.6) | 1.03(1.00,1.06) |  | 26.7(25.4,27.9) | 0.95(0.88,1.03) |  | 13.2(12.2,14.1) | 1.00(0.88,1.15) |
| **Q8:(Sleeping time) how long did you sleep every day before classes resumed during the COVID-19 epidemic?(Converting according to sleep standard questionnaire)** | | | | | | | | | | |
| Positive: | Enough | 6,226 | 57.6(56.4,58.8) | Ref. |  | 26.8(25.4,28.3) | Ref. |  | 13.8(12.5,15.0) | Ref. |
| Negative: | Lack | 4,324 | 63.7(62.3,65.1) | 1.11(1.07,1.14) |  | 29.0(27.1,31.0) | 1.08(0.99,1.18) |  | 13.0(11.6,14.4) | 0.95(0.82,1.09) |
| **Family and subjective factors** | | | | | | | | | | |
| **Q1: (Father myopia) Does your father suffer from myopia?** | | | | | | | | | | |
| Positive: | No | 10,275 | 56.6(55.8,57.5) | Ref. |  | 25.3(24.2,26.5) | Ref. |  | 12.7(11.7,13.7) | Ref. |
| Negative: | Yes | 3,988 | 68.8(67.4,70.1) | 1.21(1.18,1.24) |  | 33.1(30.8,35.4) | 1.31(1.20,1.42) |  | 14.1(12.6,15.6) | 1.11(0.98,1.27) |
| **Q2: (Mother myopia) Does your mother suffer from myopia?** | | | | | | | | | | |
| Positive: | No | 9,880 | 56.2(55.3,57.1) | Ref. |  | 24.5(23.4,25.7) | Ref. |  | 12.4(11.4,13.4) | Ref. |
| Negative: | Yes | 4,383 | 68.6(67.3,69.8) | 1.22(1.19,1.25) |  | 34.5(32.4,36.7) | 1.41(1.30,1.52) |  | 14.5(13.1,16.0) | 1.17(1.03,1.33) |
| **Q3:(Selfperceived vision loss) During the COVID-19 epidemic when classes are not resumed, have you experienced a sudden loss of vision in the last 5 months?** | | | | | | | | | | |
| Positive: | No | 6,624 | 22.1(20.9,23.3) | Ref. |  | 9.7(8.3,11.1) | Ref. |  | 12.4(11.4,13.4) | Ref. |
| Negative: | Yes | 5,115 | 43.4(40.7,46.2) | 1.97(1.81,2.14) |  | 14.5(13.4,15.6) | 1.50(1.27,1.77) |  | 14.5(13.1,16.0) | 1.17(1.03,1.33) |
| **Q4:(Selfperceived eyestrain) During the COVID-19 epidemic when classes are not resumed, did you suddenly feel that your eyes are getting tired or dry?** | | | | | | | | | | |
| Positive: | No | 7,463 | 54.8(53.8,55.8) | Ref. |  | 24.5(23.3,25.8) | Ref. |  | 11.5(10.3,12.8) | Ref. |
| Negative: | Yes | 5,499 | 67.5(66.3,68.8) | 1.23(1.20,1.27) |  | 32.6(30.4,34.7) | 1.33(1.22,1.45) |  | 14.7(13.5,15.9) | 1.27(1.11,1.46) |
| **Q5:(Sitting posture remind) During the COVID-19 epidemic when classes are not resumed,have your parents or teachers often reminded you that your reading and writing posture is incorrect?** | | | | | | | | | | |
| Positive: | Yes | 11,801 | 58.8(57.9,59.6) | Ref. |  | 27.1(26.0,28.2) | Ref. |  | 13.0(12.1,13.9) | Ref. |
| Negative: | No | 2,434 | 66.4(64.5,68.3) | 1.13(1.09,1.17) |  | 27.2(24.5,29.9) | 1.00(0.90,1.11) |  | 13.8(11.9,15.6) | 1.06(0.91,1.23) |
| **Q6:(Desks brightness) Do you feel the desktop light on your study desk is bright enough before classes resumed during the COVID-19 epidemic?** | | | | | | | | | | |
| Positive: | Enough | 10,081 | 58.1(57.1,59.1) | Ref. |  | 26.4(25.3,27.6) | Ref. |  | 13.1(12.1,14.1) | Ref. |
| Negative: | No | 4,025 | 65.4(64.0,66.9) | 1.11(1.07,1.14) |  | 29.4(27.3,31.4) | 1.09(1.00,1.19) |  | 13.5(12.0,14.9) | 1.02(0.90,1.17) |
| **Q7:(Seat comfort level) Do you think the height of your study desk and chair is suitable for your height before classes resumed during the COVID-19 epidemic?** | | | | | | | | | | |
| Positive: | Yes | 12,416 | 59.3(58.5,60.2) | Ref. |  | 27.3(26.2,28.3) | Ref. |  | 13.2(12.3,14.1) | Ref. |
| Negative: | No | 1,221 | 65.1(62.4,67.8) | 1.11(1.06,1.16) |  | 26.6(22.8,30.3) | 0.92(0.79,1.07) |  | 12.6(10.1,15.1) | 0.95(0.78,1.18) |
| Note: The myopia prevalence, incidence and progression rates were calculated by adjusting for age, sex, province, provincial socioeconomic levels, and urban/rural areas. | | | | | | | | | | |

**In order to further verify the reliability and validity of the questionnaire, we conducted a household survey to collect the questionnaire again in three provinces (Jiangsu, Shanghai, and Chongqing) two weeks after the questionnaire survey. Our investigators adopted the method of household survey, in which 240 households in three provinces were randomly selected by the project team for face-to-face questionnaire investigation. The same questionnaire towards children was collected again in household. The questionnaire recovery rate was 100% in the household investigation. The reliability and validity of each question in the questionnaire were assessed using the correlation coefficient of the two survey response options. The correlation coefficient of retest reliability of each factor question was above 0.50. The Cronbach’s alpha, a standard measure of internal consistency evaluating the reliability of the questionnaire, of the overall questionnaire was 0.655, and for each of three sections was 0.527 for eye use habits factors, 0.664 for lifestyle factors and 0.671 for family and subjective factors, respectively.**

| **Table S2.** The details of questionnaires reliability and validity of the questionnaire with 23 myopia influencing factors and their three sections | |
| --- | --- |
| **Questions** | **Retest reliability*** |
|  |  |
| **Eyeuse habits factors** | **0.527#** |
| Q1: (Online courses) Do you need to take online classes (computer, tablet or mobile phone, etc) every day when classes were not resumed during the COVID-19 epidemic? | **0.844** |
| Q2:(Online courses time) The total amount of time you spent online during the COVID-19 epidemic without resuming classes? (Calculating according to the curriculum) | **0.717** |
| Q3:(Online courses break) If classes are not resumed during the COVID-19 epidemic, will there be a break during each online class? | **0.500** |
| Q4:(Overlooking habit) Do you have the habit of looking far away during the online courses breaks | **0.547** |
| Q5:(Screen time) How much time have you spent watching TV, computer, mobile phone and tablet (including surfing the Internet, watching movies, surfing the Web and playing games) every day during the COVID-19 epidemic? | **0.557** |
| Q6:(Video games time) How much time per day have you spent playing video games during the COVID-19 epidemic? | **0.999** |
| Q7:(Lying down watching) Have you been reading a book or watching a video (tablet or phone) while lying down during the COVID-19 epidemic? | **0.613** |
| Q8:(Dark watching) Have you been often looking at your phone or tablet after turning off the lights during the COVID-19 epidemic? | **0.746** |
| **Lifestyles factors** | **0.664#** |
| Q1: (MVPA time) When classes did not resume during the COVID-19 epidemic, how much time did you spend doing moderate to high intensity physical exercise per day per week (7 days)? (Converting according to standard questionnaire) | **0.814** |
| Q2:(Walking time) What is the average amount of walking time (and at least 10 minutes at a time) per day per week (7 days) during the COVID-19 epidemic when classes are not resumed? | **0.882** |
| Q3:(Sedentary time) During the COVID-19 epidemic when classes are not resumed,how much time per day did you spend on sedentary time? | **0.580** |
| Q4:(Outdoor time) What was your daily exposure to sunlight during the epidemic before classes resumed?(Time spent in direct sunlight) | **0.547** |
| Q5:(Sweets eating) How many times a week did you eat sweets (including candies, cakes, chocolates, sweet soups, etc.) before classes resumed during the COVID-19 epidemic? | **0.659** |
| Q6:(SSBs drinking) How many times a week did you drink sugar-sweetened beverages (including Cola, iced tea, nutrition express, etc.) before classes resumed during the COVID-19 epidemic? | **0.699** |
| Q7:(Fried food eating) How many times a week did you eat fried food (such as deep-fried dough sticks, oil cakes, French fries, Fried chicken wings, etc.) before classes resumed during the COVID-19 epidemic? | **0.630** |
| Q8:(Sleeping time) how long did you sleep every day before classes resumed during the COVID-19 epidemic?(Converting according to sleep standard questionnaire) | **0.768** |
| **Family and subjective factors** | **0.671#** |
| Q1: (Father myopia) Does your father suffer from myopia? | **0.999** |
| Q2: (Mother myopia) Does your mother suffer from myopia? | **0.999** |
| Q3:(Selfperceived vision loss) During the COVID-19 epidemic when classes are not resumed, have you experienced a sudden loss of vision in the last 5 months? | **0.512** |
| Q4:(Selfperceived eyestrain) During the COVID-19 epidemic when classes are not resumed, did you suddenly feel that your eyes are getting tired or dry? | **0.514** |
| Q5:(Sitting posture remind) During the COVID-19 epidemic when classes are not resumed,have your parents or teachers often reminded you that your reading and writing posture is incorrect? | **0.552** |
| Q6:(Desks brightness) Do you feel the desktop light on your study desk is bright enough before classes resumed during the COVID-19 epidemic? | **0.658** |
| Q7:(Seat comfort level) Do you think the height of your study desk and chair is suitable for your height before classes resumed during the COVID-19 epidemic? | **0.580** |
| *Test-retest reliability coefficient. # Cronbach's Alpha. | |

| **Table S3.** The myopia prevalence in 2019 and 2020 survey, and incidence in each subgroup during the COVID-19 epidemic | | | | |
| --- | --- | --- | --- | --- |
| Groups | 2019 Prevalence | 2020 Prevalence | 2019-2020 Incidence | P Values |
| **Sexes** |  |  |  |  |
| Boys | 45.4(44.2,46.5) | 56.9(55.8,58.1) | 25.3(23.9,26.6) | <0.001 |
| Girls | 51.1(49.9,52.3) | 63.1(62.0,64.2) | 29.2(27.7,30.7) |  |
| **Areas** |  |  |  |  |
| Urban | 52.5(51.4,53.6) | 63.9(62.8,64.9) | 28.4(27.0,29.9) | 0.009 |
| Rural | 42.8(41.6,44.1) | 55.1(53.9,56.4) | 25.7(24.3,27.2) |  |
| **Regions** |  |  |  |  |
| Jiangsu | 56.5(54.2,58.9) | 68.4(66.2,70.7) | 13.6(11.9,15.2) | <0.001 |
| Shanghai | 52.7(50.3,55.1) | 68.2(65.9,70.4) | 15.5(13.8,17.3) |  |
| Henan | 54.4(51.8,57.0) | 63.7(61.2,66.2) | 12.8(11.0,14.5) |  |
| Fujian | 51.5(49.2,53.8) | 62.7(60.5,64.9) | 13.4(11.8,14.9) |  |
| Guangxi | 49.5(47.3,51.7) | 60.6(58.4,62.7) | 13.6(12.1,15.1) |  |
| Chongqing | 49.2(46.9,51.6) | 60.1(57.8,62.4) | 14.0(12.4,15.7) |  |
| Gansu | 46.1(43.7,48.5) | 58.6(56.2,61.0) | 14.7(13.0,16.4) |  |
| Shanxi | 32.1(29.6,34.5) | 47.1(44.5,49.8) | 16.1(14.1,18.0) |  |
| Hunan | 33.2(30.2,36.2) | 40.3(37.2,43.4) | 12.3(10.2,14.4) |  |
| **Ages** |  |  |  |  |
| 7 | 9.5(8.2,10.8) | 22.9(21.0,24.8) | 18.7(16.9,20.6) | <0.001 |
| 8 | 18.1(16.1,20.1) | 35.5(33.0,37.9) | 24.7(22.2,27.2) |  |
| 9 | 24.5(22.3,26.8) | 44.2(41.6,46.8) | 28.9(26.2,31.6) |  |
| 10 | 36.6(34.1,39.1) | 52.5(49.9,55.1) | 29.5(26.5,32.4) |  |
| 11 | 48.9(46.4,51.5) | 63.4(60.9,65.9) | 32.6(29.2,36.0) |  |
| 12 | 58.5(55.8,61.1) | 69.5(67.1,72.0) | 32.0(28.1,35.9) |  |
| 13 | 64.8(62.2,67.3) | 74.2(71.8,76.5) | 31.3(27.1,35.5) |  |
| 14 | 72.8(70.3,75.3) | 79.5(77.2,81.8) | 31.8(26.8,36.9) |  |
| 15 | 83.1(79.0,87.3) | 85.9(82.1,89.8) | 32.2(25.8,38.7) |  |
| 16 | 82.7(80.4,84.9) | 86.8(84.9,88.8) | 32.3(25.7,38.9) |  |
| 17 | 84.5(82.5,86.6) | 88.3(86.5,90.1) | 32.8(26.0,39.6) |  |
| 18 | 79.3(72.8,85.9) | 88.7(83.5,93.8) | 51.6(33.0,70.2) |  |
| **Total** | 48.2(47.4,49.1) | 60.0(59.2,60.8) | 27.1(26.1,28.1) |  |

| **Table S4.** The myopia grade and progression rates in each subgroup during the COVID-19 epidemic | | | | |
| --- | --- | --- | --- | --- |
| Groups | Myopia Grade and Progression | | | |
|  | Mild | Moderate | Severe | Progression rates |
| **Sexes** |  |  |  |  |
| Boys | 32.2(31.1,33.3) | 19.0(18.1,19.9) | 5.2(4.7,5.8) | 12.7(11.5,13.8) |
| Girls | 35.5(34.4,36.7) | 21.7(20.7,22.6) | 5.5(4.9,6.0) | 13.6(12.5,14.7) |
| **Areas** |  |  |  |  |
| Urban | 35.1(34.1,36.2) | 22.0(21.1,22.9) | 6.1(5.6,6.7) | 13.0(11.9,14.0) |
| Rural | 32.3(31.2,33.5) | 18.3(17.3,19.2) | 4.4(3.9,4.9) | 13.5(12.2,14.8) |
| **Regions** |  |  |  |  |
| Jiangsu | 35.4(33.1,37.7) | 25.5(23.4,27.6) | 7.2(6.0,8.4) | 11.4(9.4,13.5) |
| Shanghai | 35.6(33.3,38.0) | 24.7(22.6,26.8) | 6.9(5.7,8.2) | 12.9(10.7,15.2) |
| Henan | 36.6(34.1,39.1) | 22.2(20.1,24.4) | 4.4(3.3,5.5) | 12.9(10.5,15.2) |
| Fujian | 31.8(29.6,33.9) | 23.0(21.0,24.9) | 7.4(6.2,8.7) | 17.2(14.7,19.6) |
| Guangxi | 35.2(33.1,37.3) | 21.3(19.5,23.1) | 3.9(3.0,4.8) | 12.4(10.3,14.5) |
| Chongqing | 34.0(31.8,36.3) | 20.0(18.1,21.9) | 6.0(4.9,7.1) | 13.7(11.4,16.0) |
| Gansu | 34.5(32.2,36.8) | 18.2(16.4,20.1) | 5.5(4.3,6.6) | 13.4(10.9,15.8) |
| Shanxi | 32.3(29.8,34.8) | 10.5(8.9,12.1) | 3.4(2.4,4.3) | 13.0(9.8,16.1) |
| Hunan | 26.2(23.4,29.1) | 12.3(10.2,14.4) | 1.5(0.7,2.3) | 8.4(5.3,11.5) |
| **Ages** |  |  |  |  |
| 7 | 21.3(19.4,23.1) | 1.4(0.8,1.9) | 0.2(0.0,0.3) | 6.8(3.1,10.6) |
| 8 | 30.9(28.4,33.3) | 3.9(2.9,4.9) | 0.4(0.1,0.8) | 12.9(8.8,17.1) |
| 9 | 37.5(35.0,40.1) | 5.8(4.6,7.0) | 0.5(0.1,0.9) | 12.2(8.8,15.7) |
| 10 | 39.6(37.0,42.1) | 11.5(9.8,13.1) | 0.8(0.3,1.2) | 15.4(12.3,18.6) |
| 11 | 41.1(38.5,43.6) | 19.1(17.1,21.2) | 2.3(1.5,3.0) | 15.9(13.2,18.7) |
| 12 | 41.6(39.0,44.3) | 23.3(21.0,25.6) | 4.0(3.0,5.1) | 15.7(13.1,18.2) |
| 13 | 39.4(36.8,42.0) | 29.2(26.8,31.7) | 5.2(4.0,6.4) | 15.0(12.6,17.4) |
| 14 | 36.6(33.9,39.3) | 33.5(30.8,36.2) | 9.1(7.5,10.7) | 13.7(11.4,16.0) |
| 15 | 29.7(24.6,34.7) | 43.2(37.7,48.7) | 12.9(9.2,16.6) | 11.0(7.2,14.8) |
| 16 | 27.0(24.4,29.6) | 42.8(39.9,45.7) | 16.9(14.7,19.1) | 10.4(8.4,12.4) |
| 17 | 28.1(25.5,30.7) | 41.8(39.0,44.6) | 18.3(16.1,20.5) | 10.2(8.3,12.1) |
| 18 | 29.1(21.7,36.5) | 46.6(38.5,54.8) | 12.8(7.4,18.3) | 17.1(14.2,20.0) |
| **Total** | 33.5(32.7,34.3) | 20.1(19.4,20.8) | 5.3(4.9,5.7) | 13.2(12.4,14.0) |

| **Table S5 (1/3)**. The change of adjusted myopia prevalence, incidence and progression rates and its risks ratios by different comprehensive scores groups based on 23 comprehensive scores groups | | | | | | | | |
| --- | --- | --- | --- | --- | --- | --- | --- | --- |
| Eyeuse habit factors | Group 1 | |  | Group 2 | |  | Group 3 | |
|  | Adjusted Prevalence | ARR |  | Adjusted Incidence | ARR |  | Adjusted Progression rates | ARR |
| 1 Online courses(yes) | |  |  |  |  |  |  |  |
| No | 56.4(53.1,59.6) | Ref. |  | 25.9(21.4,30.5) | Ref. |  | 14.0(10.7,17.3) | Ref. |
| Yes | 58.6(56.5,60.8) | 1.04(0.98,1.10) |  | 26.4(24.3,28.4) | 1.02(0.86,1.20) |  | 13.2(11.6,14.8) | 0.94(0.73,1.21) |
| 2 Online courses duration(≥5h) | |  |  |  |  |  |  |  |
| <5h | 57.3(52.4,62.3) | Ref. |  | 26.7(24.4,29.0) | Ref. |  | 13.4(11.8,15.1) | Ref. |
| ≥5h | 80.4(75.6,85.2) | **1.40(1.29,1.53)** |  | 28.6(19.1,38.1) | 1.07(0.76,1.51) |  | 12.2(9.5,14.9) | 0.91(0.72,1.14) |
| 3 Online courses break(yes) | |  |  |  |  |  |  |  |
| Yes | 58.2(56.3,60.2) | Ref. |  | 26.8(25.7,27.9) | Ref. |  | 13.3(12.4,14.1) | Ref. |
| No | 58.7(54.2,63.2) | 1.01(0.94,1.08) |  | 27.5(24.2,30.9) | 1.03(0.90,1.17) |  | 11.8(9.0,14.6) | 0.89(0.70,1.13) |
| 4 Overlooking habit(yes) | |  |  |  |  |  |  |  |
| Yes | 55.2(50.8,59.7) | Ref. |  | 27.0(25.8,28.2) | Ref. |  | 13.6(12.6,14.7) | Ref. |
| No | 64.7(68.8,70.5) | **1.17(1.12,1.23)** |  | 26.7(24.8,28.7) | 0.99(0.91,1.08) |  | 12.4(11.1,13.7) | 0.91(0.80,1.04) |
| 5 Screen time(≥2.5h) | |  |  |  |  |  |  |  |
| <2.5h | 51.9(47.6,56.1) | Ref. |  | 26.0(24.8,27.2) | Ref. |  | 13.2(12.0,14.3) | Ref. |
| ≥2.5h | 68.4(63.0,73.8) | **1.32(1.24,1.41)** |  | 29.4(27.5,31.3) | **1.13(1.04,1.23)** |  | 13.3(12.0,14.5) | 1.01(0.88,1.15) |
| 6 Video games time(≥4h) | |  |  |  |  |  |  |  |
| <4h | 57.7(52.7,62.6) | Ref. |  | 26.7(24.1,28.5) | Ref. |  | 13.5(12.0,15.1) | Ref. |
| ≥4h | 68.1(61.7,74.4) | **1.18(1.10,1.27)** |  | 29.2(23.8,34.6) | 1.11(0.93,1.33) |  | 11.6(8.3,14.8) | 0.85(0.64,1.14) |
| 7 Lying down watching(yes) | |  |  |  |  |  |  |  |
| No | 51.1(46.8,55.5) | Ref. |  | 24.8(22.6,27.0) | Ref. |  | 13.2(11.4,15.1) | Ref. |
| Yes | 61.1(55.8,66.3) | **1.19(1.13,1.29)** |  | 27.0(24.6,29.3) | **1.09(1.01,1.17)** |  | 13.4(11.6,15.2) | 1.01(0.85,1.20) |
| 8 Dark watching(yes) | |  |  |  |  |  |  |  |
| No | 52.1(48.1,56.1) | Ref. |  | 25.9(23.7,28.2) | Ref. |  | 13.3(11.6,15.0) | Ref. |
| Yes | 67.0(61.9,72.1) | **1.29(1.23,1.34)** |  | 27.0(24.1,29.9) | 1.04(0.95,1.14) |  | 13.4(11.4,15.4) | 1.01(0.85,1.20) |

| **Table S5 (2/3)**.The change of adjusted myopia prevalence, incidence and progression rates and its risks ratios by different comprehensive scores groups based on 23 comprehensive scores groups | | | | | | | | |
| --- | --- | --- | --- | --- | --- | --- | --- | --- |
| Eyeuse habit factors | Group 1 | |  | Group 2 | |  | Group 3 | |
|  | Adjusted Prevalence | ARR |  | Adjusted Incidence | ARR |  | Adjusted Progression rates | ARR |
| 1 MVPA time | |  |  |  |  |  |  |  |
| ≥1h | 57.1(52.5,61.8) | Ref. |  | 27.5(25.1,29.8) | Ref. |  | 12.8(10.8,14.9) | Ref. |
| <1h | 58.5(52.8,64.3) | 1.02(0.96,1.09) |  | 27.5(24.6,30.5) | 1.00(0.90,1.11) |  | 13.7(11.9,15.6) | 1.06(0.89,1.27) |
| 2 Walking time | |  |  |  |  |  |  |  |
| ≥1h | 56.1(55.1,59.0) | Ref. |  | 26.6(23.5,29.6) | Ref. |  | 12.7(10.7,14.7) | Ref. |
| <1h | 60.7(59.4,62.0) | **1.06(1.02,1.11)** |  | 27.8(24.8,30.8) | 1.05(0.92,1.18) |  | 13.2(11.9,14.5) | 1.04(0.86,1.25) |
| 3 Sedentary time | |  |  |  |  |  |  |  |
| <5h | 52.4(47.8,56.3) | Ref. |  | 26.4(24.7,28.1) | Ref. |  | 12.2(10.5,13.9) | Ref. |
| ≥5h | 68.4(62.3,74.5) | **1.31(1.23,1.40)** |  | 31.5(29.2,33.7) | **1.19(1.08,1.31)** |  | 13.8(12.4,15.3) | 1.13(0.95,1.36) |
| 4 Sun exposure time | |  |  |  |  |  |  |  |
| ≥1h | 57.8(55.7,60.0) | Ref. |  | 25.8(23.5,28.0) | Ref. |  | 13.4(11.7,15.1) | Ref. |
| <1h | 59.7(57.6,61.8) | **1.03(1.00,1.06)** |  | 27.3(24.7,30.0) | 1.05(0.96,1.16) |  | 11.4(11.7,15.0) | 0.98(0.84,1.15) |
| 5 Sweets eating | |  |  |  |  |  |  |  |
| Never | 59.2(57.3,61.1) | Ref. |  | 26.2(22.7,29.7) | Ref. |  | 10.8(8.4,13.2) | Ref. |
| Often/occasionally | 60.2(59.3,61.1) | **1.02(0.98,1.05)** |  | 26.3(24.1,28.5) | 1.00(0.88,1.14) |  | 13.9(12.3,15.4) | **1.29(1.03,1.60)** |
| 6 SSBs drinking | |  |  |  |  |  |  |  |
| Never | 55.3(50.2,60.3) | Ref. |  | 28.0(26.1,29.8) | Ref. |  | 12.8(11.1,14.6) | Ref. |
| Often/occasionally | 59.6(54.5,64.6) | **1.08(1.03,1.13)** |  | 26.8(25.6,28.0) | 0.96(0.89,1.04) |  | 13.5(11.7,15.3) | 1.05(0.90,1.23) |
| 7 Fried food eating | |  |  |  |  |  |  |  |
| Never | 58.8(57.4,60.3) | Ref. |  | 28.1(26.3,29.9) | Ref. |  | 13.1(11.7,14.6) | Ref. |
| Often/occasionally | 60.7(59.7,61.6) | **1.03(1.00,1.06)** |  | 26.7(25.4,27.9) | 0.95(0.88,1.03) |  | 13.2(12.2,14.1) | 1.00(0.88,1.15) |
| 8 Sleeping time | |  |  |  |  |  |  |  |
| Enough | 56.8(51.7,61.9) | Ref. |  | 26.8(25.4,28.3) | Ref. |  | 13.8(12.5,15.0) | Ref. |
| Lack | 61.4(55.5,67.3) | **1.08(1.02,1.14)** |  | 29.0(27.1,31.0) | 1.08(0.99,1.18) |  | 13.0(11.6,14.4) | 0.95(0.82,1.09) |

| **Table S5 (3/3)**. The change of adjusted myopia prevalence, incidence and progression rates and its risks ratios by different comprehensive scores groups based on 23 comprehensive scores groups | | | | | | | | |
| --- | --- | --- | --- | --- | --- | --- | --- | --- |
| Eyeuse habit factors | Group 1 | |  | Group 2 | |  | Group 3 | |
|  | Adjusted Prevalence | ARR |  | Adjusted Incidence | ARR |  | Adjusted Progression rates | ARR |
| 1 Father Myopia | |  |  |  |  |  |  |  |
| No | 55.2(53.2,57.1) | Ref. |  | 24.7(22.5,26.9) | Ref. |  | 12.6(11.1,14.1) | Ref. |
| Yes | 67.2(64.7,69.7) | **1.22(1.18,1.26)** |  | 32.1(28.8,35.6) | **1.30(1.16,1.46)** |  | 15.0(12.9,17.0) | **1.19(1.05,1.35)** |
| 2 Mother Myopia | |  |  |  |  |  |  |  |
| No | 54.9(53.1,56.7) | Ref. |  | 24.1(22.2,26.0) | Ref. |  | 12.4(10.9,14.0) | Ref. |
| Yes | 66.7(63.9,69.4) | **1.22(1.18,1.25)** |  | 33.2(29.5,37.0) | **1.38(1.25,1.52)** |  | 15.1(12.9,17.3) | **1.21(1.04,1.41)** |
| 3 Self-perceived vision loss | |  |  |  |  |  |  |  |
| No | 46.3(43.8,48.7) | Ref. |  | 21.4(19.2,23.6) | Ref. |  | 9.3(7.6,11.1) | Ref. |
| Yes | 75.5(72.7,78.4) | **1.63(1.54,1.73)** |  | 41.1(37.2,45.1) | **1.92(1.69,2.19)** |  | 14.7(13.0,16.4) | **1.58(1.29,1.93)** |
| 4 Selfperceived eyestrain | |  |  |  |  |  |  |  |
| No | 53.2(50.9,55.4) | Ref. |  | 23.7(21.5,25.9) | Ref. |  | 11.6(9.8,13.3) | Ref. |
| Yes | 65.4(62.6,68.2) | **1.23(1.17,1.29)** |  | 31.3(28.1,34.5) | **1.32(1.18,1.48)** |  | 14.7(12.7,16.8) | **1.28(1.05,1.55)** |
| 5 Sitting posture remind | |  |  |  |  |  |  |  |
| No | 56.9(52.1,61.7) | Ref. |  | 27.1(26.0,28.2) | Ref. |  | 13.2(11.6,14.7) | Ref. |
| Yes | 64.4(58.6,70.2) | **1.13(1.08,1.19)** |  | 27.2(24.5,29.9) | 1.00(0.90,1.11) |  | 14.0(11.8,16.2) | 1.06(0.91,1.23) |
| 6 Desks brightness | |  |  |  |  |  |  |  |
| Enough | 56.6(51.8,61.5) | Ref. |  | 25.8(23.6,27.9) | Ref. |  | 13.1(11.3,14.9) | Ref. |
| No | 63.4(57.7,69.2) | **1.12(1.07,1.17)** |  | 28.5(25.9,31.1) | **1.10(1.02,1.20)** |  | 14.0(11.8,16.3) | 1.07(0.88,1.30) |
| 7 Seat comfort level | |  |  |  |  |  |  |  |
| Yes | 57.5(52.6,62.4) | Ref. |  | 27.3(26.2,28.3) | Ref. |  | 13.2(12.3,14.1) | Ref. |
| No | 63.9(58.4,69.3) | **1.11(1.06,1.16)** |  | 26.6(22.8,30.3) | 0.92(0.79,1.07) |  | 12.6(10.1,15.1) | 0.95(0.78,1.18) |

| **Table S6.** The change of adjusted myopia prevalence, incidence and progression rates and its risks ratios by different comprehensive scores groups based on 23 comprehensive scores groups | | | | | | | | |
| --- | --- | --- | --- | --- | --- | --- | --- | --- |
| Comprehensive scores groups | Group 1 | |  | Group 2 | |  | Group 3 | |
|  | Adjusted Prevalence | ARR |  | Adjusted Incidence | ARR |  | Adjusted Progression rates | ARR |
| ≤-5 | 68.2(64.5,71.9) | 1.15(1.06,1.25) |  | 35.1(28.6,41.7) | 1.47(1.12,1.94) |  | 13.6(11.0,16.2) | 0.82(0.59,1.12) |
| -5 to -2 | 68.0(64.4,71.5) | 1.15(1.06,1.24) |  | 33.5(28.8,38.2) | 1.40(1.09,1.82) |  | 14.9(12.0,17.9) | 0.90(0.61,1.31) |
| -2 to 0 | 65.7(62.3,69.1) | 1.11(1.02,1.20) |  | 30.3(24.2,36.5) | 1.27(0.97,1.67) |  | 16.2(12.2,20.2) | 0.97(0.65,1.45) |
| 0 | 59.2(54.6,63.8) | Reference |  | 23.9(17.6,30.1) | Reference |  | 16.7(11.9,21.5) | Reference |
| 0 to 2 | 61.6(58.1,65.0) | 1.04(0.97,1.12) |  | 29.9(26.3,33.6) | 1.25(0.95,1.66) |  | 14.2(10.5,17.9) | 0.85(0.56,1.31) |
| 2 to 5 | 58.6(56.2,60.9) | 0.99(0.91,1.07) |  | 26.1(23.0,29.2) | 1.09(0.83,1.43) |  | 13.3(10.2,16.4) | 0.80(0.56,1.13) |
| 5 to 10 | 53.5(50.7,56.3) | 0.90(0.85,0.96) |  | 24.4(21.5,27.3) | 1.02(0.81,1.29) |  | 11.5(9.3,13.6) | 0.69(0.49,0.97) |
| >10 | 52.1(49.1,55.0) | 0.88(0.81,0.95) |  | 23.3(20.9,25.8) | 0.98(0.76,1.26) |  | 8.8(5.9,11.8) | 0.53(0.33,0.84) |
| P_trend_ values | <0.001 | |  | <0.001 | |  | <0.001 | |
| Note: The adjusted myopia prevalence, incidence and progression rates and adjusted risk ratios (ARR) were calculated after adjusting for age, sex, province and urban/rural area， and the cluster effect of schools. | | | | | | | | |

| **Table S7.**  The attribution of cumulative factor and the theoretical myopia prevalence, incidence and progression rates if multiple influencing factors was eliminated | | | | | | |
| --- | --- | --- | --- | --- | --- | --- |
| Groups | Controlled Variables | Myopia Prevalence |  | Myopia Incidence |  | Myopia Progression rate |
| PAR%* | 8 Eyeuse habit factors | 16.1(12.9,19.3) |  | 4.7(1.1,8.2) |  | 0.5(-2.7,3.8) |
|  | 8 Lifestyles factors | 9.7(5.3,14.1) |  | 5.1(1.2,9.1) |  | 2.3(-1.8,6.3) |
|  | 7 Family and subjective factors | 24.7(23.4,26.0) |  | 7.5(6.3,8.7) |  | 4.6(3.1,6.1) |
|  | All 23 factors | 27.8(19.9,35.4) |  | 9.5(2.0,17.0) |  | 4.6(0.5,8.7) |
| Theoretical values# | 8 Eyeuse habit factors | 44.8(41.5,48.1) |  | 22.9(19.4,26.8) |  | 12.6(9.6,16.4) |
|  | 8 Lifestyles factors | 49.5(44.9,54.1) |  | 23.0(19.1,27.4) |  | 10.4(6.9,15.4) |
|  | 7 Family and subjective factors | 34.1(32.5,35.8) |  | 19.2(17.7,20.8) |  | 8.4(7.0,10.1) |
|  | All 23 factors | 31.3(24.0,39.8) |  | 18.1(11.6,27.1) |  | 8.3(5.0,13.7) |
| Note:* All such values are PAR% (95% confidence intervals) calculated after adjusting for age, sex, province and urban/rural area.# The theoretical myopia prevalence, incidence and progression rates (95% confidence intervals) are calculated based on the separate PAR%. | | | | | | |


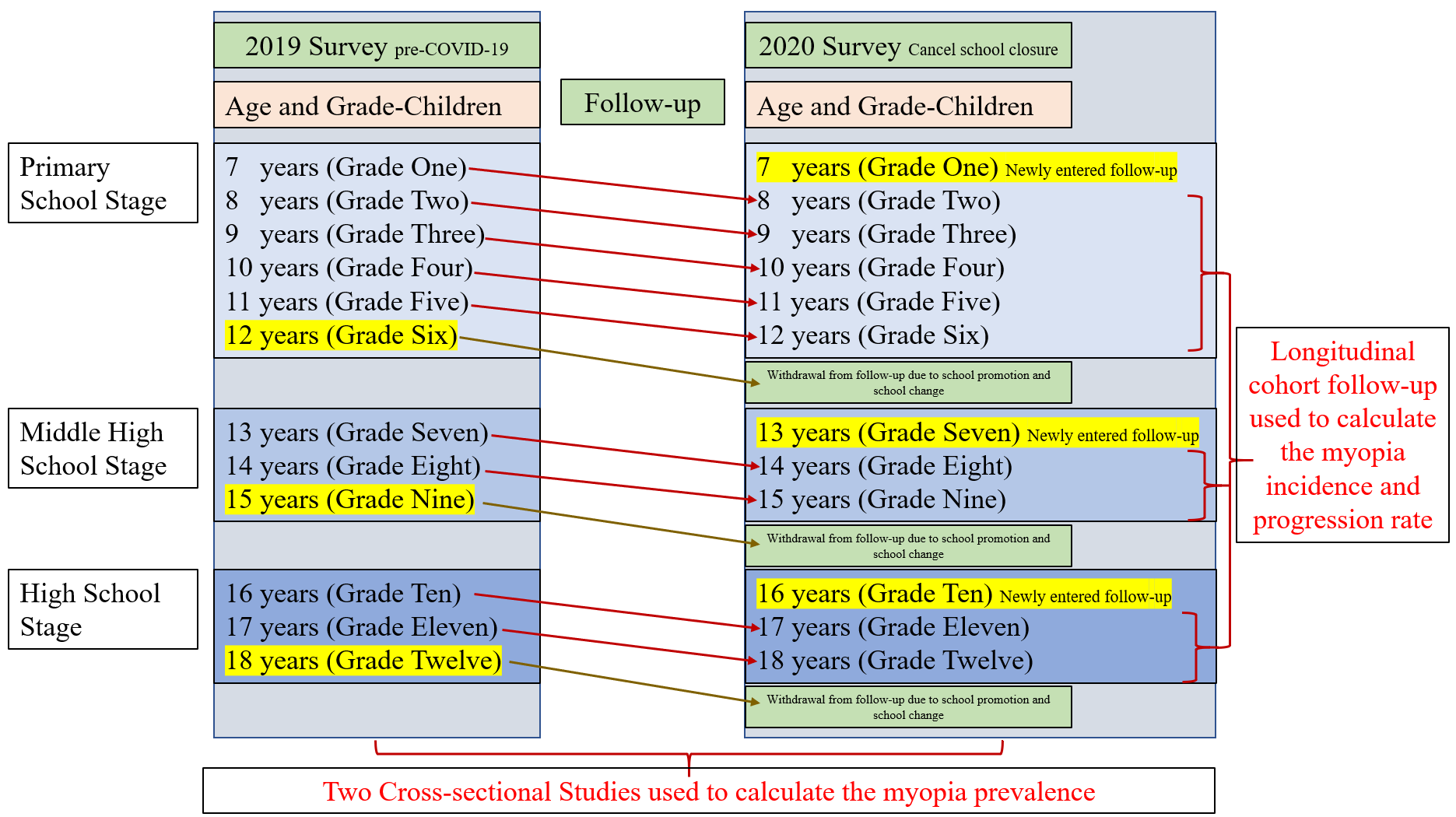


**Figure S1. Flow chart that shows the matched** **longitudinal design obtained from the two cross-sectional surveys**
